# Descriptive analysis of municipal policies addressing shade in eight southwest and northeast states in the United States

**DOI:** 10.3389/fpubh.2025.1565251

**Published:** 2025-07-14

**Authors:** David B. Buller, Alishia Kinsey, Taylor Sullivan, Phoebe Gruetter, Margaret C. Morrissey-Basler, Ian D. Buller, Carolyn J. Heckman

**Affiliations:** ^1^Research, Klein Buendel, Inc., Golden, CO, United States; ^2^Science Department, Science Park High School, Newark, NJ, United States; ^3^Department of Health Sciences, Providence College, Providence, RI, United States; ^4^Public Health and Scientific Research, DLH, LLC, Bethesda, MD, United States; ^5^Behavioral Sciences Section, Cancer Prevention and Control Program, Rutgers Cancer Institute, Rutgers University, New Brunswick, NJ, United States

**Keywords:** shade, policy, municipalities, environment, heat illness, skin cancer

## Abstract

**Introduction:**

Shade is an essential environmental feature to prevent heat illnesses and skin cancer. Written policies related to shade were described in municipalities in four southwest and four northeast U.S. states.

**Method:**

Municipal codes, planning documents, and manuals/guidelines from municipalities (*N* = 48) in eight U.S. states were coded for content related to shade by research assistants. They used a standardized protocol to assign numeric codes to each document to assess type of document, type of shade, location, resource allocation, accountability, and design standards. Results were summarized using descriptive statistics.

**Results:**

Three quarters of municipalities (75.0%) had a policy document that addressed shade, including municipal codes (54.2%), planning documents (29.2%), and manuals/guidelines (12.5%). Protecting from heat (31.3%) was mentioned in policies more than protecting from ultraviolet radiation (8.3%), as was natural shade (56.3%) rather than constructed shade (25.0%). Policies prescribed several design standards, most frequently shade material, proportion of area covered, and attractiveness. Half (50.0%) of municipalities mentioned accountability for shade in the policy, but only a third (35.4%) addressed resource allocation. Regional differences were seen in policy document type, shade type, locations, design standards, and resource allocation.

**Discussion:**

Many municipalities had policies that mentioned shade, but only a minority of policies indicated that the purpose of the policy was protection from heat or ultraviolet radiation. In northeast municipalities, which can have local home rule traditions, policies on shade appeared almost entirely in municipal codes. Southwest municipalities often included policies in planning documents that may have less legal force than municipal codes.

## 1 Introduction

Shade is an essential environmental feature for public health (1), as extreme heat events linked to climate change (2) contribute to heat illnesses (e.g., heat syncope, heat exhaustion, and heat stroke) and death (3) and acute and chronic ultraviolet radiation (UV) exposure causes persistent high skin cancer (melanoma and keratinocyte cancers) incidence and deaths (4, 5). The U.S. Centers for Disease Control and Prevention (6, 7) (CDC) recommends using shade to manage body temperature to prevent heat illnesses and to reduce solar UV exposure to prevent skin cancer (7–10). Shade has several advantages for personal protection from heat and UV: being inexpensive, requiring little planning, being attractive, enhancing comfort, and impinging minimally on outdoor activities. Shade is desired by many Americans (11–15) and may be used when provided in public open spaces (16–18).

Many adults and children in the United States may have limited access to shade, particularly in neighborhoods with racial and ethnic minorities and low-income residents (19, 20), producing environmental shade disparities. Racial and ethnic minority and low-income populations are at disproportionately high risk for heat illnesses (21, 22) and skin cancer due to lack of access to shade and other cooling methods (23, 24), low-income outdoor jobs (25), low access to dermatologists, few skin exams, late diagnosis of skin cancer (26–32), and infrequent personal sun protection (33–36).

Public policy is an important strategy for providing shade in public spaces and addressing shade equity more generally (37, 38). Policy can elevate shade on community leaders’ agendas, achieve equitable distribution of shade by neighborhood, and motivate investment in shade despite its costs. Shade policy has received little research attention in public health (20).

This paper examined written policies related to shade in municipalities in four southwest and four northeast U.S. states, using legal mapping methods that described the type of documents and content. The authors were located in these two regions and the regions had differing climates and ambient UV levels. Municipalities administer public open spaces and parks. These areas are often community-oriented, within walking distance for 75% of residents, contain venues for outdoor recreation/leisure activities [and physical activity (39–45)] that expose users to heat/UV, and are used by all age, race, ethnic, and income groups (although use may be statistically low by older individuals and Black people) (39, 43, 45–49).

## 2 Method

The WCG IRB determined that this study is not human subjects research. It did not involve human participants, so IRB approval was not required.

### 2.1 Sample of municipalities

A sample of municipalities from eight states (Arizona, Colorado, Connecticut, New Jersey, New Mexico, New York, Pennsylvania, and Utah) in two regions of the United States (i.e., southwest and northeast) (*N* = 48) were selected. Regions were selected for their proximity to the authors and differences in climate, topography, and latitude to improve generalizability. The southwest states have dry climates, many hours of sunshine, very warm to hot temperatures during summer months, and are located at low latitudes, which produce dangerously high temperatures and UV. By contrast, the northeast states have humid climates with cloud cover and intermittent precipitation. Humidity can contribute to high heat exposure. All of the states have experienced summer heat waves, and UV levels are sufficiently high in summer months to sunburn the skin even on cloudy days.

Municipalities were selected in a two-step process. First, a list of all municipalities in each state was obtained and stratified into three groups based on population size—49,999 or less, 50,000–99,999, and 100,000 or more. In the second step, the largest municipality by population was selected with certainty in each state—Phoenix, Denver, Bridgeport, Newark, Albuquerque, New York City, Philadelphia, and Salt Lake City, respectively and then to ensure representativeness within each state, one city was randomly selected from the largest group and two cities each from the two smaller groups, yielding six municipalities per state (two large, two medium, and two small population cities). This produced a sample of municipalities stratified by state and population size.

### 2.2 Municipal shade policy collection and coding

Municipal codes, planning documents, and manuals/guidelines were examined, and those containing language related to shade were collected from each municipality. These documents were located using a Google search engine and municipal websites, using the search terms, shade, policy, parks, and recreation. The full text of the policy document was obtained. Policies were initially read by a research assistant who assigned content codes using a pre-set coding protocol. The research assistant met with the first author to review and refine categories and address uncertainties in codes. A second research assistant was trained to code policies by reviewing the definitions and categories and being presented with examples. The first research assistant verified the second assistant’s policy codes, resolving disagreements through discussion. A third independent research assistant coded policy documents from three cities to estimate inter-rater reliability. Cohen’s kappa, which assesses amount of agreement beyond chance between raters in categorical measures, was 0.73 and indicated substantial agreement (50).

### 2.3 Shade policy coding protocol

A coding protocol containing five categories was developed by modifying our previous assessments of workplace and school sun safety policy and from a review of the municipal policy documents by the senior author. The categories and their codes are as follows:

i Type of document (municipal/city/zoning code, planning document [master plan, general plan, or community plan], manual/guideline).
ii Type of shade (natural [trees, other vegetation], built [shade structure, building, pavilions, shelters], not specified).
iii Location (park [urban, community, neighborhood, dog, skate, recreation], public open space/areas, playground [children’s play equipment], picnic area, sitting area/benches, work area, break/lunch area, athletic field, retail shopping area, parking area, sidewalks/pathway/pedestrian areas, entrances, streets, community plaza/courtyards, paved surfaces).
iv Resource allocation and accountability (personnel or methods for implementing or monitoring policy) for shade.
v Design standards (shade pattern, avoidance of ice/snow, tree canopy coverage, tree selection, heat sink area/island, percent of shade coverage, percent of natural shade, and climate considerations [snow or ice build-up]).

### 2.4 Statistical analysis

Given the small sample of municipalities and policy documents in just eight states (which limited statistical power for infential statistics), we calculated descriptive statistics for each coding category (counts and percentages) using MS Excel Version 16.91 (RRID:SCR_016137).

## 3 Results

### 3.1 Profile of the sample of municipalities

The *N* = 48 municipalities were located in the West and Northeast Census regions within the United States. They ranged in size from 246 to 8,258,025 residents (Arizona 670–1,643,899; Colorado 1,240–713,453; Connecticut 19,008–148,028; New Jersey 1,813–304,960; New Mexico 1980–561,368; New York 1,001–8,258,035; Pennsylvania 246–1,550,542; Utah 9,159–207,677) (51). The municipal populations across the eight states included white (3.0 to 98.0%), Hispanic (0.0 to 77.2%), and African American (0.0 to 80.6%) residents (52, 53). Maximum temperatures in the eight states in July 2024 ranged from 84.0°F (Colorado) to 99.5°F (Arizona) (54). The average maximum UV Index in June from 2006 to 2023 varied by region from 10 to 13 in the southwest states to 6 to 7 in the northeast states (55).

### 3.2 Presence of policy on shade

Our search identified policy documents with content related to shade in 77.1% of the municipalities (Table 1). The policies were present mostly in municipal codes, ordinances, and planning documents (e.g., master plans), with a small number of cities including shade policies in guideline documents.

**Table 1:** Content of policies on shade in municipalities in southwest and northeast U.S. states.

Policy information	Southwest States	Northeast States	Overall
*N* of municipalities	24	24	48
	*N* (%)	*N* (%)	*N* (%)
Policy present	19 (79.2)	18 (75.0)	37 (77.1)
Type of Policy			
Municipal/city/zoning code	6 (25.0)	20 (83.3)	26 (54.2)
Planning document [master, general, or community]	13 (54.2)	1 (4.2)	14 (29.2)
Manual/guideline	5 (20.8)	1 (4.2)	6 (12.5)
Policy content			
Purpose of policy			
Heat	8 (33.3)	7 (29.2)	15 (31.3)
UV	2 (8.3)	2 (8.3)	4 (8.3)
Type of shade			
Natural	10 (41.7)	17 (70.8)	27 (56.3)
Built	10 (41.7)	2 (8.3)	12 (25.0)
Placement of shade			
Park	17 (70.8)	6 (25.0)	23 (47.9)
Playground	8 (33.3)	1 (4.2)	9 (18.7)
Dog park	1 (4.1)	0 (0.0)	1 (2.0)
Sports area	4 (16.7)	0 (0.0)	4 (8.4)
Walking trail	3 (12.5)	0 (0.0)	3 (6.3)
Sidewalks/streets	1 (4.1)	16 (66.7)	17 (35.4)
Resource allocation for shade	13 (54.2)	4 (16.7)	17 (35.4)
Accountability for shade provision	12 (50.0)	12 (50.0)	24 (50.0)
Design standards for shade			
Size	8 (33.3)	3 (12.5)	11 (22.9)
Shade material	6 (25.0)	17 (70.8)	23 (47.9)
Surface type	2 (8.3)	0 (0.0)	2 (4.2)
Heat sink/island location	4 (16.7)	0 (0.0)	4 (8.3)
Proportion of area covered	2 (8.3)	12 (50.0)	14 (29.2)
Publicly accessible	13 (54.2)	1 (4.2)	14 (29.2)
Attractiveness	13 (54.2)	5 (20.8)	18 (37.5)
Safe use of area	11 (45.8)	0 (0.0)	11 (22.9)
Climate considerations (snow/ice)	4 (16.7)	4 (16.7)	8 (16.7)

### 3.3 Policy purpose and content

Table 1 summarizes codes for policy purpose and content. A third of the policies had the stated purpose of protecting individuals from excessive heat but very few (4 of 48 documents) specified the purpose of protecting people from UV. The majority of policies pertained to the use of natural shade, while a quarter mentioned built or constructed shade. Overall, the most common locations for shade were parks and sidewalks or streets, with playgrounds mentioned in only about a fifth of policies. Half of the policies described a procedure for assessing accountability for implementing the shade policy but only a third mentioned how resources were to be allocated for shade provision.

Many policies prescribed design standards for the shade that provided functional, aesthetic, or quality frameworks for shade (56). Shade material was specified in nearly half of the policies, while 3 out of 5 policies specified the proportion of an area to be shaded and that the shade must be publicly accessible. A third of policies described ways to make the shade attractive, but only about a quarter indicated that shade should be designed to be safe to use. Considerations related to the local climate conditions, such as not contributing to snow or ice build-up, shading locations that were known to be heat sinks or heat islands, and the type of surface under the shade, were infrequently mentioned in the policies.

### 3.4 Regional differences in shade policies

A similar number of municipalities in the southwest and northeast states had policies related to shade and only a limited number of policies mentioned heat or UV protection. Policies were most commonly written in city codes in the northeast states (83.3%) and planning documents in the southwest states (54.2%) (Table 1). However, policies in southwest municipalities mentioned built shade much more than those in northeast states, where natural shade was far more likely to be described. Also, policies in the northeast states mostly mentioned shade along sidewalks or streets; policies in southwest states designated shade for parks. The southwest states also mentioned playgrounds, sports areas, and walking trails, but the policies in the northeast states did not. The design standards differed by region, too, with more northeast municipalities having policies that described the type of shade material and proportion of shaded areas covered, while southwest municipalities had policies specifying size, public accessibility, attractiveness of shade design, and safe use of shaded areas. Climate considerations were mentioned infrequently in both the southwest and northeast states; areas with heat sinks or heat islands were only included in policy documents in southwest municipalities. More southwest municipalities had policies that addressed resource allocation than northeast municipalities, although half of municipalities in both regions had policies that placed similar moderate emphasis on accountability (how was policy implementation and effectiveness monitored) for implementation of the policy.

## 4 Discussion

Municipalities play a key role in providing safe outdoor spaces. This study sought to describe how U.S. municipalities in two regions of the United States addressed the provision of shade in public areas and if policies were designed for protection from the hazards of heat and UV. Most of the 48 municipalities examined in the American southwest and northeast had a policy document that addressed shade, although the purpose was not usually described as intended for protection from heat or UV. The low latitudes, higher elevations, higher temperatures, and more sunny days in the southwest states might lead us to expect more shade policies in that region than in northeast states. However, shade may be a desirable environmental feature in all regions for UV and heat protection and also for physical and mental health and social cohesion. The publicity focused on the rising numbers of extreme heat events (2) may explain why protection from heat was much more commonly mentioned than protection from UV. UV also may be seen as having less immediate acute harm (i.e., sunburn) than heat illness, which can be acutely life-threatening. Several childcare centers,^98,99^ schools, swimming pools, and playgrounds in the United States^84,100,101^ and Australia^102-104^ have shade policies (57–71) but this was one of the first assessments of policies at the municipal level.

Many shade policies were contained in municipal codes that had legal force, especially in the northeast states. There may be strong home rule traditions in northeast states that result in more regulatory autonomy, so there was a tendency to write shade policies in municipal codes (72). By comparison, a number of policies in southwest states were in plans and guidelines that may be more advisory or impact decision-making but have less influence on public or private actions because they do not have the force of law. Converting shade plans to ordinances or codes may make them more influential Also, it may be useful to advocate for adoption of shade policies by multiple governmental units (73), including counties, states, or special districts (e.g., for recreation), especially in regions where local autonomy for municipalities is less favored, coordination of programs among governmental units is weak, and management of land use varies. Interventions should consider other factors that can affect regulations such as land use development history, community culture, partnerships, collaborations, social capital, and governmental capacity (74), as well.

Advocating for public policy is an important strategy for addressing shade inequity observed in some municipalities (37, 38), especially given a recent estimate that deaths from extreme temperature days will increase disproportionately among Hispanic and non-Hispanic Black adults in the mid-21^st^ century (75). Individuals in lower socio-economic (SES) groups also may be a greater risk for heat-related illnesses than higher SES groups (76). Lack of access to shade has been cited as one cause of these disparities (23, 24). Adopting shade policy can elevate shade on community leaders’ agenda. Policies might require audits of existing shade to identify areas with shade disparities and direct resources to achieve equitable distribution of shade by neighborhood. Policies that identify and prescribe resource allocation may also help ensure that municipalities invest in shade to reduce inequities, despite costs of natural (planting and maintaining trees/other vegetation) and built shade (constructing shade structures). Adding shade requirements for building and development plans may be one way to share costs of addressing shade disparities with owners of private parcels. Costs of shade have received little attention except as a component in a few sun safety interventions (77–79), despite the United Nations’ recent prediction that substantial public investments will be needed for climate change adaptation (80). Cost information should be studied and shared to help with decision-making about and implementation of shade policy once it is adopted. Data on geographic distribution of heat illness and skin cancer incidence could be used to direct investment in shade in neighborhoods experiencing high health risks and disparities. Finally, many shade policies focused on natural shade (i.e., planting trees and other vegetation) that can take years to produce effective shade, so policies should focus on low-cost built shade which can provide immediate solar protection and requires low-cost maintenance.

### 4.1 Limitations

A limitation of this analysis was the focus on municipalities in just eight states in the United States. We attempted to improve representativeness and generalizability by using randomization to select municipalities, employing stratification to include cities of various sizes, from small to very large, and examining municipalities in two regions with different climates. Future research should obtain a larger sample from all regions of the United States and might consider stratifying on or assessing other factors that affect environmental (latitude for UV levels or temperature) or population (income) risk. Some municipal policies could have been missed with our online search strategies. Coverage biases might be reduced in the future by using legal research services (e.g., Municode; Westlaw) or contacting municipalities. We also focused our attention only on heat and UV protection as the purpose of policy, not on other physical and mental health or social cohesion benefits of shade. The analysis also relied on legal mapping procedures assessing the municipal codes and planning documents rather than the interpretation of policies and steps taken to implement them by city officials and employees charged with applying the policies. Finally, our analysis was descriptive, because the small sample of municipalities limited statistical power.

### 4.2 Conclusion

Shade and shade policy have received little research attention in public health (20), representing sizable practical and theoretical gaps in the literature on heat illness and skin cancer prevention. The majority of municipalities assessed had some type of policy on shade, although few were intended to protect from heat or UV, many over emphasized natural shade rather than constructed shade, and they varied in location and design standards for shade. Further research is needed on public policy and shade to make successful practical and political arguments for adopting or strengthening shade policies and investing public funds in providing shade. How shade policy affects actual shade availability has not been analyzed, nor have interventions to increase shade policy and implementation. Efforts to publicize the health benefits of shade and the public’s desire for shade in public spaces may help raise shade on public agendas for municipal decision-makers. Past research has assessed sentiment toward shade (11–15) but not whether residents support public funding for it and how shade policy affects public health outcomes. With 2024 being the warmest year on record globally (81), several prolonged extreme heat events (2), and high prevalence of heat-related illnesses and mortality (82), as well as melanoma and other skin cancers (4, 5), research and advocacy on shade and other community-based strategies to prevent heat illness and skin cancer is urgently needed.

## Data Availability

The raw data supporting the conclusions of this article will be made available by the authors, without undue reservation.
